# Circular RNAs: A Novel Player in Development and Disease of the Central Nervous System

**DOI:** 10.3389/fncel.2017.00354

**Published:** 2017-11-08

**Authors:** Lili Xie, Mao Mao, Kun Xiong, Bing Jiang

**Affiliations:** ^1^Department of Ophthalmology, The Second Xiangya Hospital, Central South University, Changsha, China; ^2^Departments of Ophthalmology and Anatomy, Institute for Human Genetics, UCSF School of Medicine, San Francisco, CA, United States; ^3^Department of Human Anatomy and Neurobiology, School of Basic Medical Sciences, Central South University, Changsha, China

**Keywords:** circular RNAs, back-splicing, microRNA sponge, central nervous system, biomarker

## Abstract

Circular RNAs (circRNAs) own unique capabilities to communicate with nucleic acids and ribonucleoproteins and are emerging as indispensable compositions of the regulatory messages encoded in the genome. Due to lack of 3′ termini, circRNAs are more resistant to degradation by exonuclease RNase R and possess greater stability than linear RNAs. Moreover, circRNAs can act as microRNA (miRNA) sponge and affect messenger RNA (mRNA) splicing and transcription. By virtue of their great stability and elaborate regulatory mechanisms of gene expression, circRNAs play important roles in certain physiological activities. The development, homeostasis and stress response of the central nervous system (CNS) depend upon precise temporal and spatial regulation of gene networks. Moreover, emerging evidence has revealed that circRNAs are spatiotemporally regulated and dynamically expressed during brain development; therefore, they can exert significant influences on CNS development and diseases. In this review, we highlight the biogenesis of circRNAs and their central roles in regulation of CNS development and diseases.

## Introduction

Recent technical advances in next generation sequencing have greatly accelerated discoveries in the field of molecular genetics, among which the importance of non-protein coding transcripts have been increasingly highlighted (Sharma et al., 2016). In contrast to messenger RNAs (mRNAs), the term non-coding RNAs (ncRNAs) refers to a class of RNA transcripts that do not encode protein (Hirose et al., 2014). It was estimated that only 1–2% RNA in human genome could code for proteins and ncRNAs constitute a vast majority of the human transcriptome (Dunham et al., 2012). In addition to microRNAs (miRNAs), small interfering RNAs (siRNAs), long non-coding RNAs (lncRNAs) and small nuclear RNAs (snoRNAs), the recently recognized circular RNAs (circRNAs) represents a special type of ncRNAs that plays a central regulatory role in RNA metabolism (Hentze and Preiss, 2013).

As early as in the 1970s, several circRNAs were found in RNA viruses (Sanger et al., 1976; Hsu and Coca-Prados, 1979). However, they have long thought to be byproducts of mRNA splicing until recent technical advances of deep-sequencing revealed the existence of thousands of circRNAs in difference species (Liang et al., 2017; Reddy et al., 2017; Zhao W. et al., 2017). CircRNAs represent a novel class of widespread and diverse endogenous RNAs that, unlike linear RNAs, are characterized by a covalent bond linking the 5′ and 3′ ends (Guo et al., 2014). CircRNAs are generated from spliceosome-mediated precursor mRNA (pre-mRNA) splicing through a non-canonical splicing process in eukaryotes (Ashwal-Fluss et al., 2014; Chen, 2016). Many circRNAs originate from protein coding genes and contain exonic sequences; however, they have long been considered as RNAs that do not encode any proteins (Guo et al., 2014; You et al., 2015). Nevertheless, recent studies have shown that a subset of circRNAs can be translated endogenously (Pamudurti et al., 2017; Yang et al., 2017). Due to the absence of 3′ termini, circRNAs are resistant to degradation by exonuclease RNase R and thus being more stable than linear mRNAs (Chen, 2016). CircRNAs are evolutionarily conserved across multiple species (Jeck et al., 2013; Rybak-Wolf et al., 2015) indicating their functional importance. Given their abundance, long half-life and species conservation, circRNAs may effectively regulate a wide range of physiological activities and pathological changes (Zhang X.O. et al., 2016; Zheng et al., 2016). Although the function of circRNAs remains largely uncharacterized, recent studies have shown that some circRNAs can bind other ncRNAs or proteins and thus regulating the expression of other or even their parental genes (Abdelmohsen et al., 2017; Han et al., 2017). Moreover, recent discoveries of translated products from certain circRNA also suggest that they may have specific biological functions (Pamudurti et al., 2017; Yang et al., 2017).

Multiple recent studies have shown that circRNAs may play crucial roles in brain development and diseases. In a study testing the abundance of circRNAs in different organs, mammalian brain was found to have the highest amount of circRNAs among all tissues tested, including liver, lung, heart, and testis (You et al., 2015). In addition, circRNAs are especially highly enriched in synapses, and genes related to synapses can give rise to significantly abundant circRNAs (Earls et al., 2014; Rybak-Wolf et al., 2015; Szabo et al., 2015). Interestingly, the expression levels of certain circRNAs can be much higher than the canonical linear transcripts of their parent genes during the central nervous system (CNS) development (Rybak-Wolf et al., 2015), indicating that circRNAs may have different functions than their linear counterparts. It is known that other kinds of ncRNAs, such as miRNAs play an essential role in the process of CNS development and diseases (Hill and Lukiw, 2016; Persengiev et al., 2011; Yin et al., 2015). CircRNAs can serve as competing endogenous sponges to miRNAs and therefore by regulating their activities, circRNAs can have notable influence upon the development and diseases of the CNS (Taulli et al., 2013; Lu and Xu, 2016; Li et al., 2017).

In this review, we describe the biogenesis and characteristics of circRNAs. Their potential functions and possible roles in CNS development and diseases are also discussed.

## Biogenesis of circRNAs

CircRNAs are derived from non-canonical splicing of precursor mRNAs (pre-mRNAs), which are regulated by the spliceosome and modulated by *cis*-regulatory elements and *trans*-acting factors (Zhang et al., 2013; Guo et al., 2014; Barrett and Salzman, 2016). Through RNA sequencing of non-polyadenylated transcriptomes, thousands of circRNAs were detected in different types of cells and tissues, and overall, they comprise over 10% of the transcripts encoded by their parent genes. Although the same gene contains sequences of both circRNAs and linear protein-coding mRNAs, the expression levels of circRNAs and their linear counterparts do not always correlate, indicating the presence of a distinct regulation mechanism of biosynthesis between the circRNA and linear mRNA (Jeck and Sharpless, 2014; Kelly et al., 2015; Thomas and Sætrom, 2014).

### Canonical Spliceosomes Mediate Non-canonical circRNA Biogenesis

**FIGURE 1:**
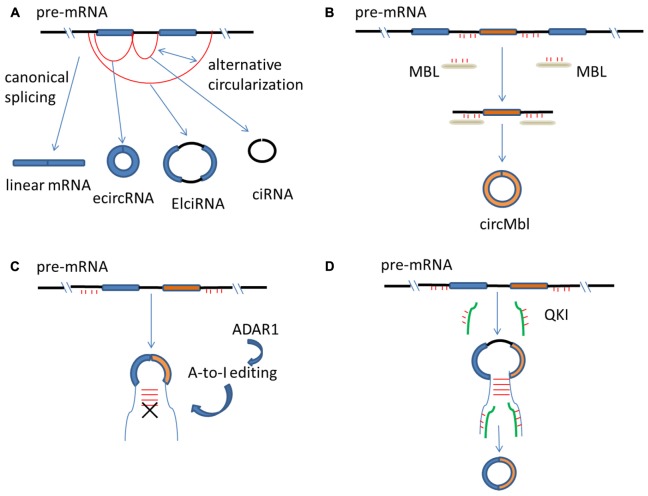
Regulation of circRNA biogenesis. **(A)** mRNAs are generated from spliceosome-mediated canonical splicing, but circRNAs are generated from pre-mRNA splicing through a non-canonical splicing process. CircRNAs can arise from exons (called ecircRNA), from introns (called ciRNA) or from both exons and introns (called ElciRNA). **(B)** The flanking introns of circMbl have abundant MBL binding sites that are required for circMbl biogenesis. Therefore, MBL protein can control the production of the circRNA of its own. **(C)** ADAR1 converts Adenosine-to-Inosine (A-to-I), and then, inhibits circRNA biosynthesis by breaking RNA–RNA interactions that form during RNA base-pairing. **(D)** The presence of QKI binding sites in the intron of genes is able to trigger circRNA production. mRNA, messenger RNA; circRNA, circular RNA; pre-mRNA, precursor mRNA; ecircRNA, exonic circRNA; ciRNA, intronic circRNA; ElciRNA, exon–intron circRNA.

CircRNAs can arise from different regions of gene loci in eukaryotes, such as from exons (called exonic circRNA, or ecircRNA), from introns (called intronic circRNA, or ciRNA) or from both exons and introns (called exon–intron circRNA, or ElciRNA) (Li Z. et al., 2015; Zhang Y. et al., 2016) (**Figure 1A**). Nevertheless, most circRNAs are derived from exons of protein-coding genes through a non-canonical splicing process called ‘back-splicing,’ in which a downstream splice donor joins an upstream splice acceptor leading to exon circularization (Jeck et al., 2013; Salzman, 2016). The canonical splice machinery, however, is required for exon circularization as was shown by mutagenesis experiments (Chen and Yang, 2015; Starke et al., 2015). Since mRNAs that are generated from linear splicing is also mediated by the canonical splicesome, how circRNAs and mRNAs can both be generated from the same pre-mRNA remains to be an important question (Hamid and Makeyev, 2014). Similar to mRNA splicing, the presence of canonical splice sites of bracketing exons can determine, to a large degree, the circularization rates (Barrett et al., 2015; Wang and Wang, 2015; Zhang Y. et al., 2016). On the other hand, functional studies have elucidated that, during circular vs. linear splicing, different splice sites can be activated and the biogenesis of circRNAs and mRNAs may compete with each other (Tay et al., 2014; Barrett and Salzman, 2016). Moreover, there are other differences between canonical spliceosome mediated circRNA and mRNA biogenesis, which might largely determine which molecule will be predominantly generated from the pre-mRNA, leading to distinct expression patterns of circRNA and mRNA (Ashwal-Fluss et al., 2014; Starke et al., 2015). For example, the efficiency of back-splicing is lower than those of the corresponding linear counterparts, perhaps because spliceosomes are inefficiently aggregated at back-splicing sites at the steady-state of circRNA production (Ashwal-Fluss et al., 2014; Guo et al., 2014). Although the exact details on how the splicesome carry out exon circularization is not known, the canonical splice signals and canonical spliceosomal machinery are important in the processing of circRNAs biogenesis (Chen and Yang, 2015; Petkovic and Müller, 2015).

### *Cis*- and *Trans*- Regulators of circRNA Biogenesis

In addition to the splicing machinery, both *cis*-regulatory elements and *trans*-acting factors are needed to bring the two splice sites into close proximity to facilitate back-splicing (Jeck and Sharpless, 2014; Kramer et al., 2015). For example, reverse and complementary sequences resided in flanking introns of circulated exons are able to form RNA duplexes, which enhance the efficiency of back-splicing (Ivanov et al., 2015; Zhang et al., 2014). RNA duplexes can form by repetitive sequences such as *Alu* elements (Kim et al., 2004; Jeck et al., 2013), as well as from non-repetitive but complementary sequences (Zhang et al., 2014). Longer complementary sequences in general have stronger RNA pairing strengths and enhanced circRNA biogenesis than shorter sequences; however, sequences as short as 30 nts also possess the ability to stimulate circRNA production (Liang and Wilusz, 2014). In addition, distinct ways of RNA pairing can lead to generation of different circRNAs. For example, repetitive sequences such as *Alu* elements can form different combinations of *Alu* pairs, which results in production of diverse circRNAs from a single gene – a process known as alternative circularization (Jeck et al., 2013) (**Figure 1A**). The types and expression levels of circRNAs are much higher in humans than in mice, and the increased circRNA complexity during species evolution is correlated with an increased number and RNA-pairing capability of those complementary sequences (Dong et al., 2016). Importantly, in addition to RNA base-pairing formed across flanking introns, RNA base-pairing can also form within an individual intron in linear RNA production (Hossain et al., 2011; Zhang et al., 2014). Therefore, there are competitions between non-canonical splicing and canonical splicing, based on choice of RNA base-pairing in flanking introns or within an individual intron (Jeck et al., 2013; Hamid and Makeyev, 2014).

*Trans*-acting factors such as RNA-binding proteins (RBPs) have been reported to be capable of regulating circRNA biogenesis (Castello et al., 2012; Conn et al., 2015; Dudekula et al., 2016). Three factors, muscleblind (MBL), Adenosine deaminae 1 acting on RNA (ADAR1) and quaking (QKI) have been shown so far to regulate circRNA production by different mechanisms (Ashwal-Fluss et al., 2014; Ivanov et al., 2015; Conn et al., 2015). MBL is a conserved regulator of RNA splicing. The second exon of MBL can be circularized to produce a circular transcript (called circMbl) and circMbl and its flanking introns have abundant MBL binding sites (Ashwal-Fluss et al., 2014). These sites are required for circMbl biogenesis and therefore, MBL protein can control the production of the circRNA of its own (Ashwal-Fluss et al., 2014) (**Figure 1B**). ADAR1 expression is negatively correlated with production of certain circRNAs (Szabo et al., 2015). ADAR1 converts Adenosine-to-Inosine (A-to-I), which predominantly occurs in double-stranded *Alu* repeats (Athanasiadis et al., 2004). Therefore, it is thought that ADAR1 inhibits circRNA biosynthesis by breaking the RNA stem of RNA-RNA interactions that form during RNA base-pairing (Ivanov et al., 2015) (**Figure 1C**). QKI regulates the production of many circRNAs and the presence of QKI binding sites in the intron of genes that are normally spliced in a linear fashion is sufficient to trigger circRNA production (Conn et al., 2015) (**Figure 1D**).

It was reported that circRNA production occurs post-transcriptionally, since a 3′ end processing signal is necessary for back-splicing and the 3′ polyadenylation is required for splicing of some introns (Liang and Wilusz, 2014). Nevertheless, other studies point out that circRNA biogenesis can occur both co-transcriptionally and post-transcriptionally, and the strength of base-pairing of complementary sequences in the flanking intronic repeats determines whether back-splicing occurs co- or post-transcriptionally (Wilusz and Sharp, 2013; Ashwal-Fluss et al., 2014; Kramer et al., 2015). While back-splicing mediated by minimal intronic repeats [<40 nucleotides (nt)] requires 3′ end processing and occur post-transcriptionally, long flanking repeats (∼400 nt) are able to produce circRNAs co-transcriptionally (Kramer et al., 2015).

## CircRNAs in CNS Development

Central nervous system development is a complex and orderly process that requires precise regulation of proliferation and differentiation of progenitor cells and is intricately controlled by gene networks (Gailite et al., 2015; Villaescusa et al., 2016). Expression of circRNAs during CNS development is tissue-specific and stage-associated (Venø et al., 2015; Dou et al., 2016; van Rossum et al., 2016). In addition, circRNAs were reported to be preferentially back-spliced from neural genes and were widely and dynamically expressed in the brain. Therefore, circRNAs might play important roles in various processes of CNS development (Venø et al., 2015; You et al., 2015; Hanan et al., 2016; Filippenkov et al., 2017).

### CircRNAs in Progenitor Cell Proliferation and Neural Differentiation

Proliferation and differentiation of neurons from stem cell precursors and progenitors are landmarks of neural development. NcRNAs such as miRNAs and lncRNAs have been shown to be important in these processes (Rani et al., 2016). Similarly, circRNAs have also become increasingly recognized to play crucial roles. During neural development, the expression pattern of neuronal circRNAs show conservation between mouse, pig, and human, and the highly expressed circRNAs is more conserved than average, indicating that these highly expressed circRNAs are potentially more important (Westholm et al., 2014; Rybak-Wolf et al., 2015) than others. In cell culture models of neural differentiation, the expression of the majority of circRNAs was shown to be significantly up-regulated (Rybak-Wolf et al., 2015). The host genes of these upregulated circRNAs are known to play essential roles in neuronal activities such as dendritic mRNA transport and synaptic membrane exocytosis (Ng et al., 2013; Heraud-Farlow and Kiebler, 2014; Jung et al., 2015). In addition, during porcine brain development, different anatomic regions were found to have different circRNA expression levels, with the minimum amount in the brain stem and the highest amount in the cortex and cerebellum (Venø et al., 2015). Moreover, at different stages of neural differentiation, there are distinct levels and types of up-regulated circRNAs (Rybak-Wolf et al., 2015). Taken together, circRNA expression is spatio-temporally regulated during brain development, suggesting that they may play an essential role in the process of neural proliferation and differentiation (Rybak-Wolf et al., 2015; Dang et al., 2016).

How do circRNAs control progenitor cell proliferation and neural differentiation? The origin of circRNAs may reflect their functions to some extent, assuming circRNAs have similar biological function to their host genes (Rybak-Wolf et al., 2015; Venø et al., 2015). In a study investigating spatio-temporal expression patterns of circRNAs during porcine embryonic brain development, three host gene associated pathways, Wnt signaling, axon guidance and the TGFβ signaling pathway, were found to be overrepresented at a developmental stage corresponding to major neurogenesis (Venø et al., 2015). These over-represented signaling pathways play a major role in neural stem cell proliferation, migration and differentiation (Rosso and Inestrosa, 2013; Mattar and Cayouette, 2014). How circRNAs impact these pathways remains to be an important question in understanding their contributions to neural proliferation and differentiation.

### CircRNAs in Synaptogenesis

The expression change, localization and origin of circRNAs signify the importance of circRNAs in synaptogenesis to a large degree. During porcine and mouse brain maturation, the amount of circRNAs produced was found to be significantly increased, especially when synaptic connections and neural network forms (Venø et al., 2015; You et al., 2015). For example, in mice, the expression pattern of most circRNAs changes abruptly at postnatal day (P)10 when synaptogenesis begins (You et al., 2015). Moreover, circRNAs originated from synapse-related genes are consistently up-regulated during the establishment of mature neural networks (You et al., 2015). In addition, consistent with a fourfold increase of synaptic density in the human cortex compared to mice (Herculano-Houzel, 2009), higher levels of circRNAs were detected in human brains compared to mouse brains. Moreover, instead of being equally distributed in all neuronal compartments, circRNAs are highly enriched in synaptoneurosomes (You et al., 2015). Although both circRNAs and mRNAs can be detected in the cell body and dendrites, they are not co-localized. For example, circStau2a is mainly presented in synapses; however, the linear Stau2a mRNA transcripts primarily locate in the cytoplasm (Venø et al., 2015). Furthermore, neural circRNAs are disproportionally originated from host genes encoding proteins with synaptic functions. Genes related to synapse, synapse part, presynaptic active zone, presynaptic membrane and postsynaptic density can give rise to abundant circRNAs (Venø et al., 2015; You et al., 2015). For example, circHomer1_a, a circRNA originated from *Homer1* that encodes a key protein in postsynaptic density regulation (Meyer et al., 2014), has the highest expression level during synaptic plasticity (You et al., 2015).

The function of circRNAs in mammalian brain remains elusive. Because of their primary localization to synapses, it has been hypothesized that circRNAs may function as topologically complex platforms which recruit ribonucleoprotein (RNP) granules and transport required RNAs and proteins (Rybak-Wolf et al., 2015) that are needed for synaptogenesis when synaptic connections forms (You et al., 2015). Moreover, due to their great stability and specificity, circRNAs might be used as synaptic tags to keep a molecular memory (Rybak-Wolf et al., 2015). Another possible function for circRNAs is to serve as miRNA sponges (Hansen et al., 2013). Certain circRNAs have been shown to contain multiple binding sites for core regions of miRNAs (Hansen et al., 2013; Zheng et al., 2016). It is possible that circRNAs can serve as competitive inhibitors for miRNA activities by inhibiting binding of miRNAs to their target transcripts. Multiple studies have shown that miRNAs play an important role in synapse formation, stabilization and plasticity (Rajasethupathy et al., 2009; McNeill and Van Vactor, 2012). Therefore, circRNAs may indirectly control synaptogenesis through regulating activities of synapse-related miRNAs and the expression of miRNA targeted mRNAs (Hansen et al., 2013; Zheng et al., 2016).

## CircRNAs in CNS Diseases

CircRNAs play essential role in various processes of CNS development and might impact the function of aging brain (Westholm et al., 2014; Gruner et al., 2016), Recently, circRNAs have been investigated in neurological diseases (Chen and Schuman, 2016; Shao and Chen, 2016; Li et al., 2017). In addition, circRNAs have shown great potential to serve as prognostic biomarkers of many diseases due to their stability, specificity, sensitivity, and conservation (Memczak et al., 2015; Rybak-Wolf et al., 2015; Lyu and Huang, 2016).

### CircRNAs in the Pathogenesis of CNS Diseases

**FIGURE 2:**
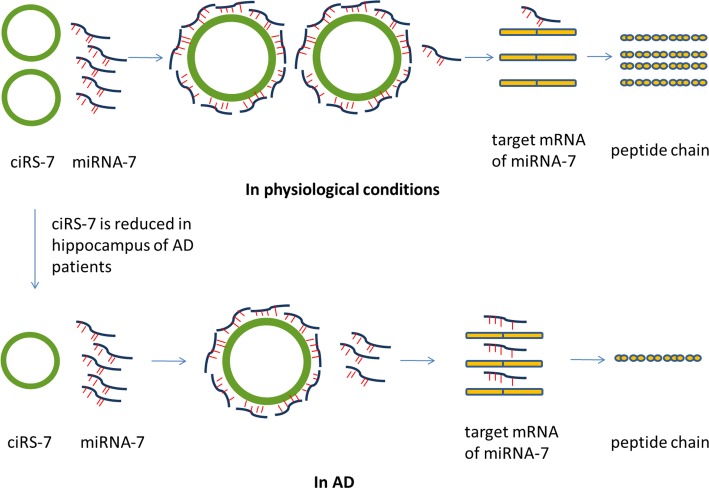
Possible role of ciRS-7 in AD. CiRS-7 contains more than 70 selectively conserved anti-miRNA-7 sequences, thus acting as competing endogenous sponges to efficiently quench normal miRNA-7 activities. However, the expression of ciRS-7 is significantly reduced in hippocampus of AD patients. The abnormal miRNA-circRNA system might result in the abnormal mRNA expression of the target genes of miRNA-7. Therefore, the abnormal circRNA-miRNA-mRNA system may underline the disease mechanism of AD. miRNA, microRNA; circRNA, circular RNA; ciRS-7, circRNA for miRNA-7; AD, Alzheimer’s disease.

Several circRNAs were shown to function as miRNA sponges that sequester miRNAs and affect their interaction with downstream mRNAs (Floris et al., 2017; Piwecka et al., 2017) and an abnormal circRNA-miRNA-mRNA system has been indicated in the pathogenesis of CNS diseases (Li et al., 2017; Zhou et al., 2017). For example, Alzheimer’s disease (AD), a progressive neurodegenerative disorder, is caused by damage to synapses, neuronal cell bodies and axons due to the accumulation of pathological amyloid-β or hyper-phosphorylated Tau in the brain (Cohen et al., 2015). miRNA-7 is abundant in the brain and can directly regulate the expression of AD-related genes such Ubiquitin protein ligase A, which mediates the clearance of amyloid peptides in AD (Zhao et al., 2016). A circRNA, ciRS-7 (circRNA for miRNA-7), also termed as cerebellar degeneration-related protein 1 antisense RNA (Cdr1as), contains more than 70 selectively conserved anti-miRNA-7 sequences, thus acting as competing endogenous sponges to efficiently quench normal miRNA-7 activities (Hansen et al., 2013). miRNA-7 and ciRS-7 were found to be co-expressed in cultured primary murine neurons and in mouse brain sections (Hansen et al., 2013). In addition, expression of ciRS-7 is significantly reduced in hippocampus of AD patients (Lukiw, 2013), suggesting an abnormal miRNA-circRNA system may underline the disease mechanism of AD (**Figure 2**). Because of the importance of miRNA-7 in other diseases such as cancer and Parkinson’s disease (Junn et al., 2009; Saydam et al., 2011; Liu et al., 2014), it is possible that ciRS-7 may exert a regulatory role in brain cancer and other neurodegenerative diseases as well. Deleting the ciRS-7 locus in mice leads to dysfunctional synaptic transmission and abnormal neuropsychiatric-like behavior in mice (Piwecka et al., 2017). Other than miR-7, ciRS-7 also has a binding site to miR-671. Interestingly, the expression of miR-7 and miR-671 were both deregulated in all brain regions in ciRS-7 deficient mice; however, the direction of changes was opposite. It was thought that the binding site on ciRS-7 is completely complimentary to miR-671, and the interaction of these two molecules could lead to AGO mediated ciRS-7 slicing and miR-671 degradation. In contrast, the binding sites for miR-7 are partially complimentary which cannot trigger ciRS-7 cleavage, leading to stabilized miR-7. Therefore, it is possible that circRNAs can serve as a platform to store and transport certain miRNAs. Similarly, another circRNA generated from the gene *Sry*, has 16 putative binding sites to miR-138 and can attenuate activity of miR-138 in culture (Hansen et al., 2013). Expression of miR-138 was increased in cell culture models of AD suggesting the role of circRNA Sry in the disease pathology (Wang et al., 2015).

Other functions of circRNA in the CNS may be indicated by their host genes. For example, expression and complexity of circRNAs peak at embryonic day (E)60 during porcine brain development when dramatic morphological changes occur in the cortex. The top three highly expressed circRNAs at E60 are circCSPP1, circHDAC2 and circRIMS2 (Venø et al., 2015). The host genes of these circRNAs are crucial for CNS development and synaptic formation (Akizu et al., 2014). For instance, CSPP1 plays a part in the normal physiological functions of primary cilia and mutations in this gene lead to a developmental brain disorder called Joubert syndrome (Tuz et al., 2014). HDAC2 is important for neuronal survival and forebrain-specific deletion of this gene can lead to excessive repetitive behaviors in mice (Mahgoub et al., 2016). RIMS2 are involved in synaptic vesicles release and presynaptic plasticity and deletion of this gene can result in decreased activation of the presynaptic Ca^2+^-channel (Kaeser et al., 2012). CircRNAs might also play a role in nerve damage. Biosynthesis of circHomer1 may compete with synthesis of Homer1b/c mRNA (You et al., 2015). In an *in vitro* model inducing synaptic plasticity using primary hippocampal neurons, circHomer1a expression was remarkably increased, which could inhibit potential overexpression of Homer1b/c that is detrimental to synaptic plasticity (You et al., 2015). Down-regulation of Homer1b/c was also shown to improve neuronal survival in an *in vitro* traumatic nerve injury model (Fei et al., 2014). Therefore, circHomer1a may have great potential as a therapeutic target on repair and regeneration of injured nerves.

### CircRNAs as Potential Biomarkers in CNS Disorders

Due to lack of 3′ termini, circRNAs are generally endowed with a strong resistance to exonuclease RNase R, thus being more stable than linear RNAs (Memczak et al., 2013; Enuka et al., 2016). The great stability and abundance of circRNAs make them as great candidates for molecular biomarkers in neurological diseases. Recently, several studies have reported the use of circRNAs as potential diagnostic biomarkers for atherosclerotic vascular disease, pre-diabetes and varieties of tumors including gastric cancer, hepatocellular carcinoma, lung cancer, colon carcinoma, laryngeal cancer and leukemia, et al (Ren et al., 2016; Xie et al., 2016; Zhao Z. et al., 2017). For example, circPVT1 could act as independent prognostic marker in gastric cancer (Chen J. et al., 2017). The expression of circPVT1 was up-regulated in gastric cancer tissues and it could promote cell proliferation through serving as a sponge for miR-125 family members (Chen J. et al., 2017). Also, similarly, circular RNA hsa_circ_0000190 was down-regulated in both human gastric cancer tissues and plasma samples and had higher sensitivity and specificity compared with traditional gastric cancer biomarkers, carcinoembryonic antigen (CEA) and CA19-9 (Chen S. et al., 2017). So far, multiple circRNAs have been explored as a biomarker for a variety of diseases especially in cancer (Meng et al., 2017).

Neural circRNAs show high conservation during evolution and are abundantly expressed in the brain (Venø et al., 2015). In Drosophila, circRNA expression increased substantially during CNS aging and could be regarded as a class of aging biomarkers (Westholm et al., 2014). Accumulation of circRNAs in aging brains has also been described in mice (Gruner et al., 2016) suggesting it could be a general theme for other species including humans. Therefore, although it has not been reported in CNS diseases, because circRNAs are stable, specific, conserved and sensitive, distinct circRNA profiles may be used to evaluate the physiologic and pathological circumstances in the brain (Memczak et al., 2013; Rybak-Wolf et al., 2015; Song et al., 2016). On the other hand, biopsies for neuronal tissues may be difficult to access. However, the pathological information of CNS tissues could be acquired through detecting the levels of certain components in the plasma and cerebrospinal fluid, etc. that are easily accessible (Luo et al., 2013). Therefore, detecting circRNAs from these sources can be possible and using circRNAs as biomarkers for neurological diseases should be further investigated.

**FIGURE 3:**
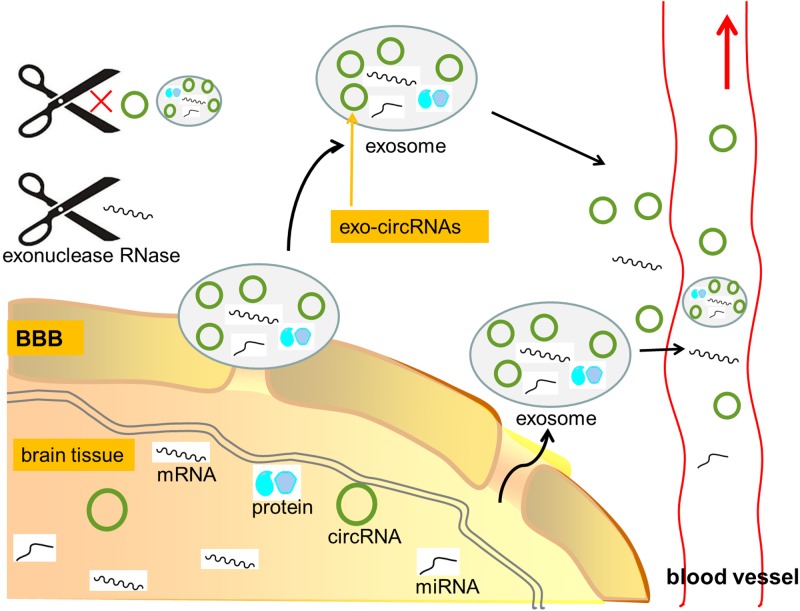
CircRNAs as potential biomarkers in CNS disorders. Due to lack of 3′ termini, circRNAs are generally endowed with a strong resistance to exonuclease RNase R, thus being more stable than linear RNAs. Furthermore, in CNS diseases, the blood–brain barrier (BBB) can be compromised and small molecules including circRNAs and exosomes can be transported out of BBB. CircRNAs contained in exosomes (exo-circRNAs) were about twice more than those in their producer cells. CircRNAs tend to be more accessible to exosomes than linear RNAs, and the ratio of exo-circRNAs to their linear transcripts in exosomes is about six times higher than that in their producer cells. In addition, exo-circRNAs showed high stability, perhaps because of their protein partners and the protection of exosomes. Thus, the great stability and abundance of circRNAs make them as great candidates for molecular biomarkers in neurological diseases. mRNA, messenger RNA; circRNA, circular RNA; miRNA, microRNA; BBB, blood–brain barrier; exo-circRNAs, circRNAs contained in exosome.

Furthermore, in CNS diseases, the blood–brain barrier (BBB) can be compromised and small molecules including circRNAs and exosomes can be transported out of BBB (Grapp et al., 2013). Exosomes are membranous small vesicles that can transfer circRNA, miRNA, mRNA and protein into extracellular fluid or cells (Cocucci and Meldolesi, 2015). Recent studies revealed that, circRNAs contained in exosomes (exo-circRNAs) were about twice more than those in their producer cells (Li Y. et al., 2015). CircRNAs tend to be more accessible to exosomes than linear RNAs, and the ratio of exo-circRNAs to their linear transcripts in exosomes is about six times higher than that in their producer cells (Li Y. et al., 2015) (**Figure 3**). In addition, exo-circRNAs showed high stability, perhaps because of their protein partners and the protection of exosomes (Li Y. et al., 2015). Taking together, the specific circRNAs profiles carried in exosomes might serve as potential biomarkers in CNS disorders (Lu and Xu, 2016).

## Future Exploration

Despite of recent discoveries that significantly expanded our knowledge on circRNAs (Danan et al., 2012; Kumar et al., 2016; Floris et al., 2017), there are still questions remain to be answered regarding circRNA biogenesis and their biological functions. These questions include: (i) how the spliceosome acts during the course of back-splicing reaction and the exact kinetics of back-splicing are currently not well known. (ii) In addition to acting as miRNA and RBP sponges, circRNAs could transport required RNAs and proteins and recruit ribonucleoproteins. However, we do not know enough details about the topological structure of circRNAs and how they interact with ribonucleoproteins and other RNAs. (iii) Neural circRNAs are abundant, conserved and spatio-temporally regulated. However, what controls the selectively high expression of certain neural circRNAs and the relative balance between the expression of circRNAs and their linear transcripts during CNS development is not known. (iv) In CNS development, how the highly expressed circRNAs act and how they are degraded are not clear. (v) Though miRNA sponge is the only known function for some circRNAs, other functions of most of circRNAs in the brain are not known. (vi) CircRNAs in certain tissues or in exosomes show high abundance and stability, but detection of their existence in exosomes can be costly. Thus, efficiency and convenient methods used for detecting circRNAs in exosomes are required for their widespread use as biomarkers. (vii) The repeatability, reliability and security of using circRNAs as biomarkers or therapeutic targets need to be further studied due to their complex roles in the body.

## Author Contributions

LX prepared an initial draft and contributed to writing all sections and preparation of the final manuscript. MM contributed to overseeing revisions and preparation of the final manuscript. KX and BJ contributed to overseeing revisions and preparation of the final manuscript.

## Conflict of Interest Statement

The authors declare that the research was conducted in the absence of any commercial or financial relationships that could be construed as a potential conflict of interest.
